# A Randomized Prospective Study of Lumpectomy Margin Assessment with Use of MarginProbe in Patients with Nonpalpable Breast Malignancies

**DOI:** 10.1245/s10434-014-3602-0

**Published:** 2014-03-05

**Authors:** Freya Schnabel, Susan K. Boolbol, Mark Gittleman, Tami Karni, Lorraine Tafra, Sheldon Feldman, Alice Police, Neil B. Friedman, Scott Karlan, Dennis Holmes, Shawna C. Willey, Moshe Carmon, Kristen Fernandez, Stephanie Akbari, Jay Harness, Lisa Guerra, Thomas Frazier, Karen Lane, Rache M. Simmons, Alison Estabrook, Tanir Allweis

**Affiliations:** 1 Department of Surgery, NYU Langone Medical Center, NYU Clinical Cancer Center, New York, NY USA; 2 Department of Surgery, Beth Israel Medical Center, New York, NY USA; 3 Department of Surgery, Breast Care Specialists, Allentown, PA USA; 4 Department of Surgery, Assaf Harofeh Medical Center, Zerifin, Israel; 5 Department of Surgery, Anne Arundel Medical Center, Annapolis, MD USA; 6 Department of Surgery, Columbia University Medical Center, New York, NY USA; 7 Department of Surgery, Pacific Breast Care, Costa Mesa, CA USA; 8 Department of Surgery, Mercy Medical Center, Baltimore, MD USA; 9 Department of Surgery, Cedars Sinai Medical Center, West Hollywood, CA USA; 10 Department of Surgery, University of South California, Los Angeles, CA USA; 11 Department of Surgery, Georgetown University Hospital, Washington, DC USA; 12 Department of Surgery, Shaare Zedek Medical Center, Jerusalem, Israel; 13 Department of Surgery, Franklin Square Hospital Center, Baltimore, MD USA; 14 Department of Surgery, Virginia Hospital Center, Arlington, VA USA; 15 Department of Surgery, St. Joseph Hospital, Orange, CA USA; 16 Department of Surgery, HOAG Hospital, Newport Beach, CA USA; 17 Department of Surgery, Bryn Mawr Hospital, Bryn Mawr, PA USA; 18 Department of Surgery, UC Irvine Medical Center, Orange, CA USA; 19 Department of Surgery, Weill Medical College of Cornell University, New York, NY USA; 20 Department of Surgery, St. Luke’s Roosevelt, New York, NY USA; 21 Department of Surgery, Hadassah Medical Organization, Jerusalem, Israel

## Abstract

**Background:**

The presence of tumor cells at the margins of breast lumpectomy specimens is associated with an increased risk of ipsilateral tumor recurrence. Twenty to 30 % of patients undergoing breast-conserving surgery require second procedures to achieve negative margins. This study evaluated the adjunctive use of the MarginProbe device (Dune Medical Devices Ltd, Caesarea, Israel) in providing real-time intraoperative assessment of lumpectomy margins.

**Methods:**

This multicenter randomized trial enrolled patients with nonpalpable breast malignancies. The study evaluated MarginProbe use in addition to standard intraoperative methods for margin assessment. After specimen removal and inspection, patients were randomized to device or control arms. In the device arm, MarginProbe was used to examine the main lumpectomy specimens and direct additional excision of positive margins. Intraoperative imaging was used in both arms; no intraoperative pathology assessment was permitted.

**Results:**

In total, 596 patients were enrolled. False-negative rates were 24.8 and 66.1 % and false-positive rates were 53.6 and 16.6 % in the device and control arms, respectively. All positive margins on positive main specimens were resected in 62 % (101 of 163) of cases in the device arm, versus 22 % (33 of 147) in the control arm (*p* < 0.001). A total of 19.8 % (59 of 298) of patients in the device arm underwent a reexcision procedure compared with 25.8 % (77 of 298) in the control arm (6 % absolute, 23 % relative reduction). The difference in tissue volume removed was not significant.

**Conclusions:**

Adjunctive use of the MarginProbe device during breast-conserving surgery improved surgeons’ ability to identify and resect positive lumpectomy margins in the absence of intraoperative pathology assessment, reducing the number of patients requiring reexcision. MarginProbe may aid performance of breast-conserving surgery by reducing the burden of reexcision procedures for patients and the health care system.

Breast-conserving surgery (BCS) has been an established approach to surgery for early-stage breast cancer for more than 30 years.^1^ Contemporary series report that 60–75 % of American women with early-stage breast cancer are treated with BCS.^2^

BCS for noninvasive and invasive cancer includes a lumpectomy procedure, with sentinel node biopsy in cases of invasive disease, and postoperative radiotherapy in most cases. A successful lumpectomy requires complete removal of the malignancy, including a margin of surrounding normal breast tissue. This can be challenging to accomplish because the microscopic extent of breast cancer can be difficult for the surgeon to discern. Multiple studies have demonstrated the association of involved or positive lumpectomy margins with an increased risk of ipsilateral breast tumor recurrence, even in the presence of radiotherapy.^3^–^6^ Although there is no universally accepted definition of negative surgical margins, at least 20 % of patients undergo more than one procedure to achieve acceptable margins as part of breast-conserving strategies.^2^,^7^,^8^

The MarginProbe (Dune Medical Devices Ltd, Caesarea, Israel) was developed to provide surgeons with real-time intraoperative assessment of lumpectomy margins. Designed to be used as an adjunct to current surgical methods, the device measures the local electrical properties (in the radiofrequency range) of breast tissue. These properties are dependent on membrane potential, nuclear morphology, and cellular connectivity and vascularity that differ between normal and malignant tissue.^9^ The device’s sensing diameter is 7 mm, and it provides a positive/negative reading for each measurement taken. The threshold for a positive reading was set based on readings directly compared to pathology results.^10^ The diagnostic performance was sensitivity 70–100 % and specificity 70–87 %, depending on the cancer feature size. The performance was similar for all histology types, including ductal carcinoma-in situ. In a multicenter trial where patients were randomized to usual surgical technique versus usual technique with adjunctive use of the MarginProbe, the rate of reexcision surgery was reduced by 56 % in the device arm of the trial.^11^ There was no difference in cosmetic outcomes.

The current study examined the contribution of adjunctive use of MarginProbe to identification of all involved lumpectomy margins, reduction in the number of patients with positive margins at the completion of primary lumpectomy surgery, and decrease in the necessity for repeat surgical procedures to achieve acceptable margins.

## Methods

This study was a prospective, randomized (1:1), double-arm, controlled trial involving 21 institutions and 53 surgeons. Participating centers represented a variety of practice settings, including academic, community-based, and private practice sites. Institutional review board approval was obtained at each site. Inclusion criteria included patients over 18 years with nonpalpable intraductal and invasive breast cancers. All patients had opted for BCS. Patients with multicentric or bilateral disease, those with prior radiotherapy or neoadjuvant chemotherapy, and those with a history of surgery in the ipsilateral breast were excluded, as were patients who were pregnant or lactating.

**Fig. 1:**
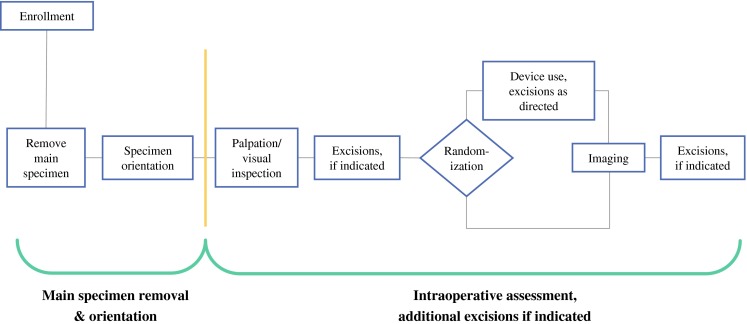
Lumpectomy procedure

Informed consent was obtained from all patients. Patients underwent preoperative localization of their lesions and removal of main lumpectomy specimens as per surgeons’ usual practices. All main lumpectomy specimens were oriented to delineate the six surfaces of the tissue (superior, inferior, medial, lateral, anterior, and posterior). After main lumpectomy specimen removal, surgeons used their usual methods of intraoperative assessment, including inspection and palpation. Intraoperative pathology assessment was precluded. If a margin was deemed to be positive or close, additional tissue was excised. Patients were then randomized to device or control arms (Fig. 1). In the control arm, surgeons completed the lumpectomies, including utilizing information from intraoperative imaging, per their routine. In the device arm, the MarginProbe was additionally used by the surgeon to examine all six surfaces of the main lumpectomy specimens, with 5–8 measurements per face. A single positive reading identified a margin as positive. Device output was recorded. Surgeons were required to excise additional tissue from the corresponding surface of the lumpectomy cavity from every device-identified positive margin. Additional tissue removed from the lumpectomy margins was not examined by the device, nor was the lumpectomy cavity. Because the device should be used within 20 min after specimen excision, device arm intraoperative imaging, with additional excisions if indicated, was performed after device use. In both study arms, main lumpectomy specimens were inked.

All specimens were evaluated by pathologists who were blinded to study arm. Tissue dimensions, margin status, and margin distance for all surfaces were recorded. Specimen volume was calculated based on the Ellipsoid formula: (π/6) × *L* × *W* × *D*. Subjects were followed (including additional surgical procedures) until the completion of surgical treatment. Data were collected until the earliest of the following events: 2 months after the patient’s last operation; conversion of the subject to mastectomy; or initiation of chemotherapy. There were no restrictions placed on surgeons in terms of the performance of additional surgical procedures. For the purposes of this study, a positive margin was considered to be disease identified at ≤1 mm from the inked edge of tissue. Diagnostic measures, including false-negative and false-positive rates, were evaluated by comparison of device readings to pathology gold standard on a margin-by-margin basis.

All statistical analyses were performed by SAS software (SAS, Cary, NC, USA). Numerical variables were tabulated using mean and standard deviations. Categorical variables were tabulated using number of observations and percentages. Statistics were performed at *α* = 0.05 two-sided significance level. Rates between arms were compared by Fisher’s exact test. Reexcisions were compared by Poisson’s regression. No missing data were imputed.

Safety was evaluated by reports of serious adverse events and adverse events. Safety reports were tabulated by group, body system, and relation to treatment.

## Results

A total of 596 patients were randomized, with 298 in each arm of the trial. Patient demographics and baseline characteristics are listed in Table 1. Patients underwent extensive imaging before surgery. The mean extent of disease was similar in the two groups. The main specimen volume was similar in both groups, reflecting no difference in surgical procedure before randomization.

**Table 1:** Patient demographics and baseline characteristics

Characteristic	Device (*n* = 298)	Control (*n* = 298)
Age, years, mean (SD)	60.3 (11.4)	60.2 (11.1)
Ethnic origin, *n* (%)		
White^a^	250 (83.9)	260 (87.2)
Black	22 (7.4)	17 (5.7)
Asian	12 (4.0)	10 (3.4)
Other	14 (4.7)	11 (3.7)
Body mass index, kg/m^2^, mean (SD)	27.9 (6.6)	28.6 (6.6)
Diagnosis, *n* (%)		
Invasive ductal	181 (60.7)	202 (67.8)
Invasive lobular	26 (8.7)	13 (4.4)
Mixed invasive	8 (2.7)	5 (1.7)
Ductal carcinoma-in situ	83 (27.9)	78 (26.2)
Receptor status, *n* (%)		
ER positive	251 (84.2)	258 (86.6)
PR positive	223 (74.8)	217 (72.8)
Preoperative imaging, *n* (%)		
Mammogram	296 (99.3)	294 (98.7)
MRI	184 (61.7)	174 (58.4)
Ultrasound	228 (76.5)	289 (97.0)
Preoperative core biopsy, *n* (%)	287 (96.3)	289 (97.0)
Mean extent of disease, cm	1.7	1.6
Main lumpectomy specimen volume, ml	61	60

*SD* standard deviation, *ER* estrogen receptor, *PR* progesterone receptor, *MRI* magnetic resonance imaging

^a^Including Hispanics

**Fig. 2:**
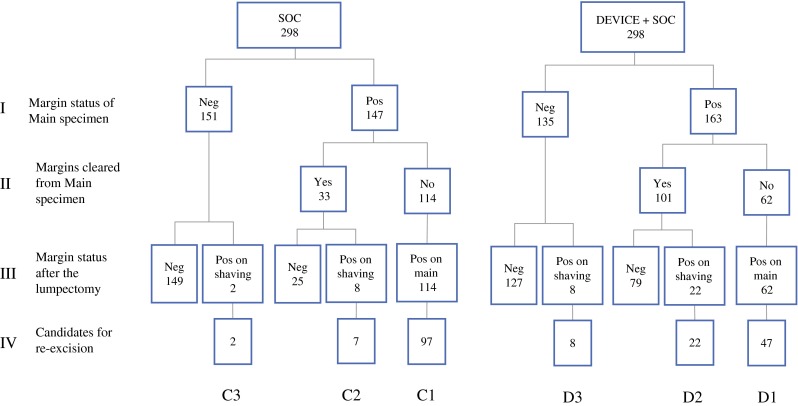
Results of intraoperative margin assessment and margin status

The disposition of patients in both arms of the trial is shown in Fig. 2. In similar proportions of patients in both arms, the main lumpectomy specimen contained at least one positive margin (Fig. 2, phase I). In patients with positive margins on initial lumpectomy specimens, an average of two margins was involved, with no difference between the two arms. With reference to the patients with positive main specimen margins, surgeons correctly identified all positive margins on the main specimen and removed additional tissue from those involved margins (Fig. 2, phase II) in 33 of 147 cases (22 %) in the control arm, versus 101 of 163 (62 %) cases in the device arm (*p* < 0.0001). Patients for whom positive margins on the main specimen were not identified remained with positive final margins after the lumpectomy (Fig. 2, phase III, branches C1 and D1). Although the main specimen was cleared, some final margins were persistently positive because of disease identified at the edge of the additional tissue resected (phase III, branches C2 and D2) in 8 and 22 cases for the control and device arms, respectively. Interestingly, additional tissue was removed from the lumpectomy cavity in both arms in cases where the main specimen was found to have clear margins, resulting in positive final margins (phase III, branches C3 and D3) in 2 and 8 patients in the control and device arms, respectively.

Table 2 lists the patients’ final margin status after the primary lumpectomy procedure. In the control arm, 41.6 % (Fig. 2, branches C1, C2, and C3) of patients had positive margins compared with 30.9 % (Fig. 2, branches D1, D2, and D3) of patients in the device arm (*p* = 0.008), representing a 26 % reduction in the positive margin rate. Even though these patients had positive margins, surgeons determined that certain patients were not candidates for reexcision because the involved margins were recorded to be at skin or fascia. Excluding these patients, the significant difference in candidates for reexcision was maintained, favoring the device arm (*p* = 0.013). More patients in the control arm were candidates for reexcision because of positive margins originating from the main specimen. In contrast, there were more candidates for reexcision in the device arm on the basis of additional cavity shavings removed.

**Table 2:** Positive margin status and reexcision lumpectomy procedures

Variable	Treatment group		Reduction	*p*
	Device (*n* = 298)	Control (*n* = 298)		
Positive margins after initial surgery				
All patients	92/298 (30.9 %)	124/298 (41.6 %)	26 %	0.008
At skin or fascia	15/298 (5.0 %)	18/298 (6.0 %)		0.72
Candidates for reexcision	77/298 (25.8 %)	106/298 (35.9 %)	27 %	0.013
Due to positive margin on main specimens	47/298 (15.8 %)	97/298 (32.9 %)	52 %	<0.001
Due to positive margin on shavings	30/298 (10.1 %)	9/298 (3.0 %)		<0.001
Reexcision lumpectomy procedures	59/298 (19.8 %)	77/298 (25.8 %)	23 %	0.097; 0.018^a^
Due to positive margin on main specimens	33/298 (10.0 %)	62/298 (20.8 %)	47 %	0.002
Due to positive margin on shavings	19/298 (7.4 %)	4/298 (1.3 %)		0.002
Due to close margins or other considerations	7/298 (2.3 %)	11/298 (3.7 %)		0.47

^a^Accounting for the difference between arms in the number of main lumpectomy specimens with positive margins (Fig. 2, phase I)

As shown in Table 2, 19.8 % of patients in the device arm underwent second procedures for reexcision of lumpectomy margins compared with 25.8 % of patients in the control arm, representing a 6 % absolute (23 % relative) reduction associated with MarginProbe use. The analysis of this difference also accounted for the small but statistically insignificant (prerandomization) difference between arms in the number of main lumpectomy specimens with positive margins (Fig. 2, phase I). With regard to reexcision procedures that were required because of positive margins originating from the main lumpectomy specimens (Fig. 2, branches C1 and D1), the control arm rate was 20.8 % compared with 10.0 % in the device arm, a 47 % reduction (*p* = 0.002).

To further evaluate device performance, the volume of tissue resected was analyzed. Both true-positive and false-positive device readings resulted in excision of additional breast tissue. Therefore, total volumes of excision were calculated across all surgeries (Table 3). As expected, the volume of tissue in main lumpectomy specimens was identical in the two arms. In the device arm, there was more tissue removed in the first surgical procedure, representing both true-positive and false-positive margin excisions. However, there was more tissue removed in reexcision procedures in the control arm. This led to an overall difference of 8.5 ml in tissue volume removed between the two study arms. When normalized to baseline breast volume, the difference between the arms was 2.6 %.

**Table 3:** Total volume of tissue removed across all surgical procedures

Characteristic	Procedures specimen volume, ml, for:		
	Device	Control	Difference
Initial surgery			
Main specimen	59.7	61.3	−1.6
Truly positive shavings	6.7	2.7	4
Falsely positive shavings	21	7.7	13
Total tissue removed (initial surgery)	87.5	71.7	15.8
Reexcision surgeries	5.8	12.8	−7
Total for all surgeries	93.3	84.8	8.5

 The performance of the MarginProbe in the provision of diagnostic information was also evaluated. The margin-level sensitivity of the device was 75.2 % (95 % CI: 69.4–81.0), with that of the control arm being 33.9 % (95 % CI: 27.6–40.2). False-negative rates were 24.8 and 66.1 % in the device and control arms, respectively. The increase in sensitivity in identification of positive margins by the device came at the expense of a reduction in margin-level specificity: 46.4 % (95 % CI: 42.9–49.9) device versus 83.4 % control (95 % CI: 81.0–85.8). False-positive rates were 53.6 and 16.6 %, in device and control arms, respectively.

Similar adverse event rates were observed in both groups: device, 6 events (2 %), and control, 5 events (2 %). Of these reports, only 1 event was possibly related to the study device (wound infection).

## Discussion

BCS is an established approach to the treatment of early-stage breast cancer, providing an equivalent outcome with mastectomy while allowing for preservation of the breast. An ongoing challenge is the requirement of negative lumpectomy margins, to reduce the risk for in-breast recurrence. There is variability in defining acceptable margin width among surgeons and radiation oncologists.^12^,^13^ Although reported reexcision rates vary, it is clear that a significant proportion of women who undergo BCS require multiple operations to achieve acceptable margins. Current techniques for intraoperative assessment have limited efficacy, particularly in cases of nonpalpable and intraductal disease.^14^–^16^ The current trial evaluated a novel device for intraoperative assessment of lumpectomy margins in a challenging population with nonpalpable disease. Adjunctive use of the MarginProbe required little additional operating time (approximately 5 min) and resulted in a statistically significant improvement in complete identification of all positive margins on main lumpectomy specimens. This study did not test whether the device would allow for less surgery to be performed if the specimens were carefully examined intraoperatively by pathologists, with or without the selective use of frozen section.

However, not all candidates for reexcision underwent these surgeries during the study period. Although 31 % of patients in the device arm had at least one positive margin at the end of the procedure, only 20 % had reexcisions. In the control arm, 42 % of patients had positive margins, and 26 % underwent reexcisions. Some patients had involved margins at skin or fascia, which are not amenable to reexcision. The design of this study did not constrain surgical decision making. The decision to perform a reexcision may be appropriately influenced by many factors, including the urgency to initiate systemic therapy, the results of genetic testing, and medical comorbidities. Although reexcision procedures were collected for 2 months after initial surgery, these factors may have had some effect on the recorded rates.

The device was designed with an emphasis on sensitivity to provide maximal detection of all positive margins. It was expected that this increase in sensitivity (decrease in false-negative results) would come at the expense of a reduction in specificity (increase in false-positive results), as was observed. The cosmetic result after BCS has multiple components and may be affected by volume of tissue excised, tumor location within the breast, size of the primary tumor, size of the breast, and postoperative radiotherapy. There is also evidence that reexcision procedures negatively affect cosmetic outcomes.^17^ Although cosmesis was not directly assessed in this study, the only factor potentially affected by MarginProbe use is volume of tissue excised. Our results suggest that use of the MarginProbe should have little impact on the cosmetic result of BCS.

Some studies have demonstrated a significant reduction in reexcision rates when additional tissue is routinely removed from all six surfaces of the lumpectomy cavity.^18^,^19^ However, a recent report from Massachusetts General Hospital showed no difference in reexcision rates in patients undergoing lumpectomy surgery, or lumpectomy plus selected or full-cavity shavings.^20^ The total tissue volume removed was smaller in the patients who underwent select or complete cavity shavings, suggesting that performance of the main lumpectomy was altered when removal of additional tissue was anticipated. This change in surgeons’ approach to the main lumpectomy specimen has also been reported in other studies.^19^ At this point, full-cavity shaving has not been widely adopted.

Achieving acceptable margins at the time of primary lumpectomy surgery may be increasingly important as techniques for intraoperative radiotherapy evolve and ablative approaches to the lumpectomy cavity are explored.^21^ When oncoplastic closure techniques are used, it is especially important to avoid positive margins. Reexcision procedures may be difficult in these cases because it can be virtually impossible to accurately identify the specific margin to be reexcised.^22^

Use of MarginProbe, as depicted in this study, is not the complete solution to the complex problem of lumpectomy margins. This device provides incremental improvement in reducing reexcision procedures, which is meaningful because these additional unanticipated procedures burden patients and the health care system. Although this device adds some additional cost, it is offset by the cost of reexcision procedures and costs related to positive margins. More work is needed to understand the relationship between various margin distances and in-breast recurrence rates. Additional evaluation of the new margins of cavity shaving specimens would also provide important intraoperative information. The use of MarginProbe or other technology to interrogate the lumpectomy cavity might provide additional data regarding the adequacy of resection. Novel methods for preoperative breast imaging might also provide a more accurate roadmap for surgical planning. The number of patients opting for mastectomy procedures is on the rise. It is possible that the frequent need for multiple excisions to achieve adequate lumpectomy margins contributes to this trend.

## Conclusion

The current study supports the use of the MarginProbe in lumpectomy surgery in the absence of routine intraoperative pathologic assessment. The device provides surgeons with intraoperative assessment of lumpectomy margins, allowing directed reexcision of positive margins and reducing the proportion of patients with positive margins at the conclusion of surgery. A decrease in reexcision procedures can reduce the burden of breast cancer surgery for the patient and the health care system.
